# Nonclinical pharmacology of daridorexant: a new dual orexin receptor antagonist for the treatment of insomnia

**DOI:** 10.1007/s00213-021-05954-0

**Published:** 2021-08-20

**Authors:** Catherine Roch, Giorgio Bergamini, Michel A. Steiner, Martine Clozel

**Affiliations:** Idorsia Pharmaceuticals Ltd, Allschwil, Switzerland

**Keywords:** Insomnia, Sleep, Orexin, Daridorexant, Dual orexin receptor antagonist, Nonclinical, Sleep architecture

## Abstract

Dual orexin receptor antagonists (DORAs) represent a novel type of sleep medication that provide an alternative to the traditionally used positive allosteric gamma-aminobutyric acid (GABA)-A receptor modulators. Daridorexant is a new DORA that exhibited in phase 3 trials in insomnia not only a beneficial effect on sleep variables, measured objectively and assessed subjectively, but also an improvement in daytime functioning. Daridorexant was discovered through a tailored research program aimed at identifying an optimized sleep-promoting molecule with pharmacokinetic properties appropriate for covering the whole night while avoiding next-morning residual activity at efficacious doses. By specific binding to both orexin receptors, daridorexant inhibits the actions of the wake-promoting orexin (also called hypocretin) neuropeptides. This mechanism avoids a more widespread inhibition of neuronal pathways and associated side effects that are intrinsic to positive allosteric GABA-A receptor modulators. Here, we review the general pharmacology of daridorexant, based on nonclinical pharmacology studies of daridorexant, unpublished or already described, or based on work with other DORAs. Some unique features of daridorexant will be highlighted, such as the promotion of natural and surmountable sleep, the preservation of memory and cognition, the absence of tolerance development or risk of physical dependence, and how it can benefit daytime functioning.

**Supplementary Information:**

The online version contains supplementary material available at 10.1007/s00213-021-05954-0.

## Introduction

Insomnia, characterized by difficulties with sleep onset and/or sleep maintenance and impairment of daytime functioning (American Academy of Sleep Medicine 2014), chronically affects approximately 5–20% of the adult population (Roth et al. 2011). Daytime impairment can negatively influence an individual’s quality of life in different manners. Daytime tiredness, distress, altered mood, and memory can significantly impair social, occupational, educational, academic, or behavioral aspects of life (Carey et al. 2005; Hudgens et al. 2020; Kyle et al. 2010a). Insomnia is associated with long-term consequences including hypertension, diabetes, and depression (Institute of Medicine (US) Committee on Sleep Medicine and Research 2006; Kyle et al. 2010b; Wilson et al. 2019).

Treatments for insomnia target gamma-aminobutyric acid type-A (GABA-A), serotonin, histamine, or melatonin receptors. Z-drugs (zopiclone, zolpidem, zaleplon), which are positive allosteric GABA-A subunit alpha 1 receptor modulators and the most widely used hypnotics, drive sleep by causing a broad inhibition of central nervous system (CNS) activity (Atkin et al. 2018; ClinCalc.com 2019). Despite their effectiveness, these medications can elicit a number of side effects, such as next-morning residual sleepiness, motor incoordination, falls, memory and cognitive impairment, and the potential of abuse, dependence, and tolerance, limiting their use (Dolder and Nelson 2008; Frey et al. 2011; Gunja 2013; Möhler 2006; Vermeeren 2004; Wafford and Ebert 2008; Wang et al. 2001; Wesensten et al. 1996; Zammit 2009).

In recent years, the orexin (also called hypocretin) system has been discovered as a target for the development of a new class of sleep medication. In 2007, our group showed for the first time that a dual orexin receptor antagonist (DORA) of the orexin type 1 and 2 receptors (OX1R, OX2R), namely almorexant, promoted sleep in rats, dogs, and healthy subjects (Brisbare-Roch et al. 2007). The orexin system and its function are thus highly conserved across species (Brisbare-Roch et al. 2007; Soya and Sakurai 2020a). In a phase 2 program in adults and elderly patients with primary insomnia, almorexant dose-dependently increased sleep efficiency, sleep time, sleep initiation, and sleep maintenance (Hoever et al. 2012; Roth et al. 2017). Since then, interest for the potential of orexin receptor antagonism in treating sleep disorders continued to grow, resulting in the US Food and Drug Administration approval of suvorexant from Merck in 2014 and lemborexant from Eisai in 2019 for the treatment of insomnia (FDA 2014, 2019).

DORAs act through an entirely different mechanism of action than the classical sleep-promoting drugs. Orexin neuropeptides are specifically expressed in a small neuronal population of the hypothalamus (de Lecea et al. 1998; Sakurai et al. 1998). Orexin neurons exert their highest activity during periods of active wakefulness and are virtually silent during sleep (Azeez et al. 2018; Gotter et al. 2013; Lee et al. 2005). They project to various wake-promoting neuronal populations, including the histaminergic neurons of the tuberomammillary nucleus (expressing mainly OX2R), the noradrenergic neurons of the locus coeruleus (expressing mainly OX1R), serotoninergic neurons of the dorsal raphe (expressing OX1R and OX2R), the dopaminergic neurons of the ventral tegmental area (expressing OX1R and OX2R), and the cholinergic neurons of the basal forebrain and the pedunculopontine and laterodorsal tegmental nuclei (expressing OX1R and OX2R) (Eggermann et al. 2001; Hagan et al. 1999; Lee et al. 2005; Liu et al. 2002; Yamanaka et al. 2002). Orexins stabilize wakefulness through the activation of these wake-promoting areas (de Lecea 2012; Sakurai 2007; Scammell et al. 2017). DORAs inhibit both OX1R and OX2R and allow sleep to occur. By selectively targeting and reducing the activity of wake-promoting neurons, the more widespread inhibition of neuronal pathways and associated side effects that are intrinsic to positive GABA-A receptor modulators can be avoided.

Daridorexant (ACT-541468) is a new DORA that successfully completed two phase 3 clinical trials in insomnia in 2020. This compound was discovered through an intensive drug discovery program on orexin receptor antagonists (Boss et al. 2020) that ensued following the discovery of almorexant (Brisbare-Roch et al. 2007). The goal was to identify a potent, dual, and brain-penetrant orexin receptor antagonist with fast onset and a duration of action, long enough for covering the night, but short enough to avoid residual activity the following morning, at optimal efficacious doses. Many promising molecules synthesized showed an increase of both rapid eye movement (REM) and non-REM sleep in rats and dogs. The choice of daridorexant among them was supported by physiology-based pharmacokinetic modeling that indicated an optimal pharmacokinetic profile for an insomnia drug in man (Boss et al. 2020; Treiber et al. 2017).

In this article, we review the nonclinical pharmacology data of daridorexant and discuss its properties and potential as a novel option for the treatment of insomnia. Since nonclinical pharmacological data are, in general, consistent for all DORAs of different chemical classes, it was not always deemed ethical to repeat certain animal experiments for daridorexant, when already performed for other DORAs. Therefore, we also discuss published nonclinical data on other DORAs, in areas in which data with daridorexant are not available, to provide a more comprehensive pharmacological overview.

## Nonclinical pharmacology of daridorexant

**Fig. 1:**
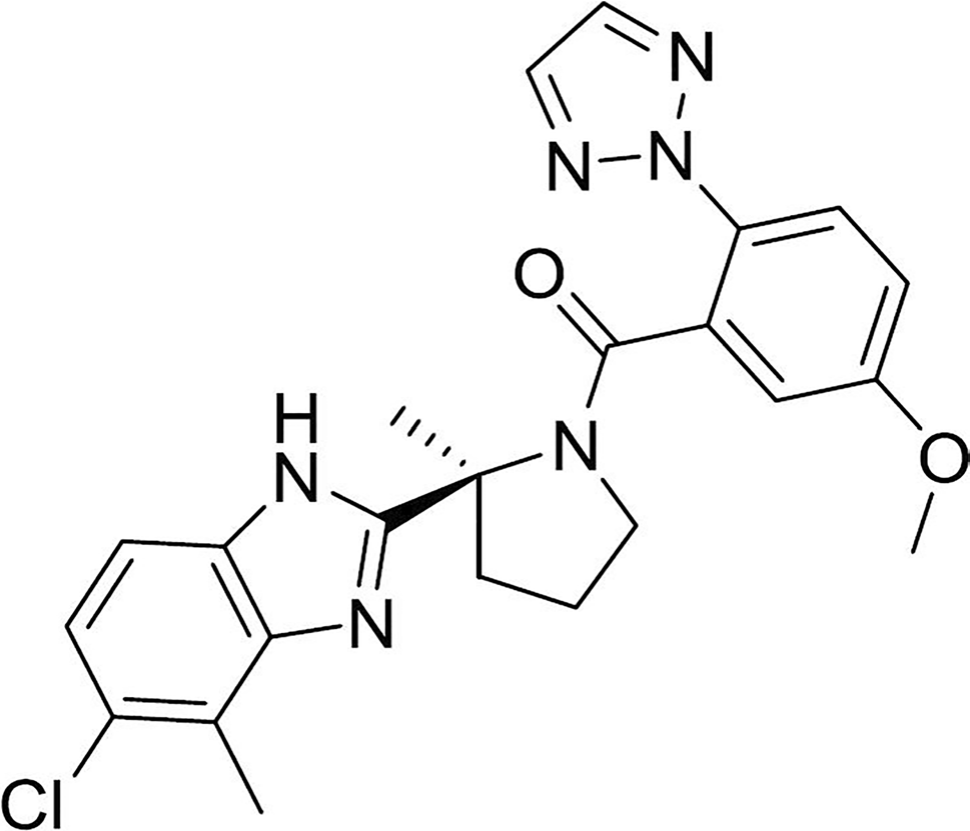
Chemical structure of daridorexant *[*(*S*)*-2-(5-chloro-4-methyl-1H-benzo[*d*]imidazol-2-yl)-2-methylpyrrolidin-1-yl](5-methoxy-2-(2*H*-1,2,3-triazol-2-yl]phenyl)methanone; also known as ACT-541468]*

Daridorexant (Fig. 1) is a potent and selective small-molecule dual OX1R and OX2R antagonist. As determined in intracellular Ca^2+^ release assays, daridorexant functions as a competitive, orthosteric antagonist with apparent *K*_*b*_ values in rat, dog, and human, respectively, of 1.1, 0.3, and 0.5 nM at OX1R and 1.7, 0.7, and 0.8 nM at OX2R (Treiber et al. 2017). Daridorexant is thus equipotent in antagonizing OX1R and OX2R. Daridorexant did not show any relevant in vitro activity in a panel screen of more than 130 central and peripheral pharmacological targets other than orexin receptors, which also included GABA receptors and other brain targets associated with abuse liability (Online Resource S1) (Treiber et al. 2017). Daridorexant is orally bioavailable in rats and dogs and effectively passes the blood–brain barrier.

### Maintenance of a natural sleep architecture

Daridorexant decreases wakefulness and thereby promotes sleep in which the architecture is preserved (Boss et al. 2020; Treiber et al. 2017). Experiments were performed in freely moving rats and dogs equipped with telemetry transmitters to record electroencephalography (EEG) and electromyography (EMG) signals. “Hyperarousal” which is thought to underlie insomnia in humans (Carskadon and Dement 2011; Levenson et al. 2015) is difficult to mimic in animals. Therefore, our experiments were conducted during the active circadian phase (night for nocturnal rats and day for diurnal dogs), when endogenous orexin levels increase (Gotter et al. 2014). Although normal wakefulness is different from hyperarousal, the good translation of the efficacy of DORAs (belonging to different chemical classes), when given during the active, night period in normal rats, to humans in clinical trials of insomnia, supports the use of such a setup for the nonclinical characterization of daridorexant.

**Fig. 2:**
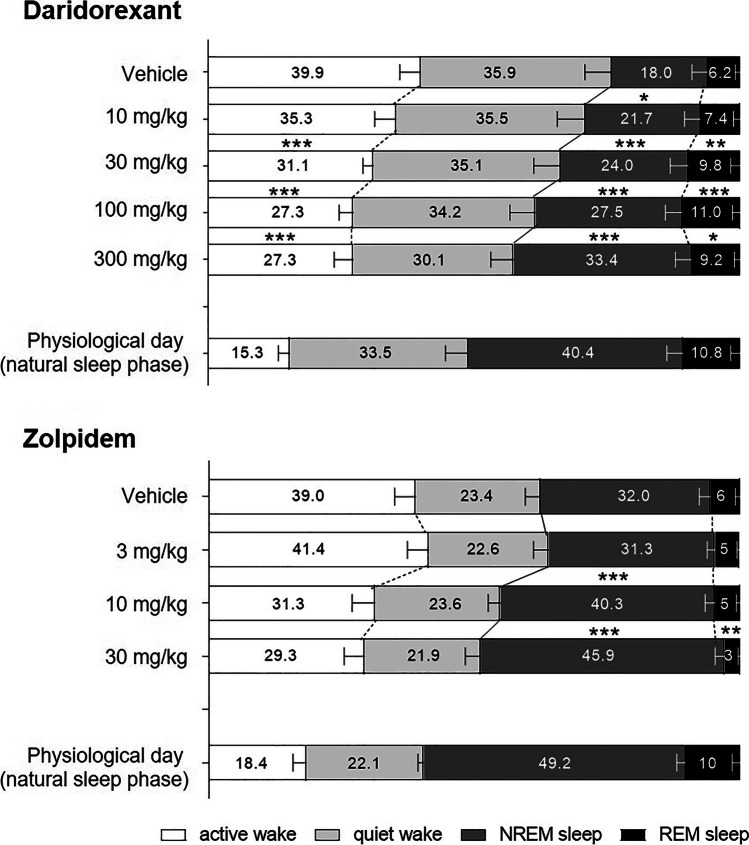
Effect of daridorexant and zolpidem on sleep and wake stages and sleep architecture during the first 6 h of the night active period in rats. Drugs were administered as single dose by oral gavage at the beginning of the dark phase. Daridorexant was formulated in a vehicle of 0.5% methyl cellulose (MC) and zolpidem in a vehicle of 0.25% MC. Values represent the percentage of time spent in each wake and sleep stage over the first 6 h of the active, night period. Physiological day values represent the first 6 h of the normal inactive, daytime period (physiological sleep period for nocturnal rats) of the respective vehicle-treated rat groups. Data are expressed as mean + standard error of the mean. One-way ANOVA was performed separately for each parameter, followed by the post hoc Dunnett’s multiple comparisons test: *p < 0.05, **p < 0.01, ***p < 0.001 vs. vehicle-treated rats. Each dose–response (daridorexant, and zolpidem) represents independent crossover studies with *n* = 8 male Wistar rats. MC, methylcellulose; REM, rapid eye movement; NREM, non-REM. ANOVA, analysis of variance. Full experimental details provided by Boss et al. 2020. Daridorexant dose response: Boss et al. The quest for the best dual orexin receptor antagonist (daridorexant) for the treatment of insomnia disorders. ChemMedChem 2020; 15:2286–2305. Copyright Wiley–VCH GmbH. Reproduced with permission. Zolpidem dose response: data-on-file

In freely moving rats, a single oral dose of daridorexant (10, 30, 100, or 300 mg/kg), administered at the beginning of their active, night phase, dose-dependently decreased active wake time and increased sleep time over the first 6-h period post-administration (Boss et al. 2020; Fig. 2). Compared to vehicle-treated rats, total wake time in daridorexant-treated rats decreased by up to 66 min, while REM and non-REM sleep time increased by up to 17 and 55 min, respectively. The time spent in REM and non-REM sleep under daridorexant treatment during the night never exceeded levels observed without drug treatment under a natural sleeping period during the day. This illustrates the natural properties of the sleep promoted by daridorexant.

Daridorexant increased REM and non-REM sleep in physiological proportions, indicating that daridorexant preserves the general sleep architecture (i.e., the non-REM/total sleep and REM/total sleep ratio) (Table 1). In contrast, in the same experimental setup, zolpidem changed the general sleep architecture. A single oral dose of zolpidem (3, 10, and 30 mg/kg) dose-dependently increased the time spent in non-REM sleep compared to vehicle-treated rats by up to 50 min but at the same time decreased the time spent in REM sleep by up to 10 min (Fig. 2, Table 1, data-on-file).

**Table 1:** Relative proportion of non-REM and REM sleep over total sleep time during the first 6 h of the night period following oral administration of daridorexant or zolpidem to rats

Daridorexant			Zolpidem		
	**% over total sleep time**			**% over total sleep time**	
	**Non-REM sleep**	**REM sleep**		**Non-REM sleep**	**REM sleep**
**Vehicle**	71.4 ± 5.6	28.6 ± 5.6	**Vehicle**	85.5 ± 1.6	14.5 ± 1.6
**10 mg/kg**	73.6 ± 3.8	26.4 ± 3.8	**3 mg/kg**	87.2 ± 1.0	12.8 ± 1.0
**30 mg/kg**	70.8 ± 4.1	29.2 ± 4.1	**10 mg/kg**	89.5 ± 1.2	10.5 ± 1.2*
**100 mg/kg**	71.7 ± 2.3	28.3 ± 2.3	**30 mg/kg**	94.0 ± 0.9	6.0 ± 0.9**
**300 mg/kg**	78.6 ± 2.1	21.4 ± 2.1			

Data are expressed as mean ± standard error of the mean. One-way ANOVA followed by the post-hoc Dunnett’s multiple comparisons test: *p < 0.05; **p < 0.001 vs. vehicle-treated rats. *N* = 8 male Wistar rats per dose group. *REM* rapid eye movement. Data-on-file. Full experimental details provided by Boss et al. 2020

Daridorexant allowed animals to fall asleep faster. The latency to the first persistent episode of both non-REM sleep (the first non-REM episode lasting ≥ 60 s) and persistent REM sleep (the first REM episode lasting ≥ 30 s) decreased in daridorexant-treated rats (Boss et al. 2020). For instance, in rats treated with 30 mg/kg daridorexant, the non-REM sleep latency decreased from 61 min (for rats treated with vehicle) to 13 min, and the REM sleep latency from 63 to 25 min.

Daridorexant showed a similar activity profile in Beagle dogs as in the rat (Boss et al. 2020). Administration of a single, oral daytime dose of daridorexant (0, 10, 30, or 90 mg/dog) dose-dependently decreased wakefulness by up to 77 min over 6 h post-administration. This was accompanied by an increase in time spent in both non-REM (up to 51 min vs. vehicle) and REM (up to 28 min vs. vehicle) sleep. Similar to the findings in rats, the ratio between the time spent in non-REM sleep (or time spent in REM) and total sleep time did not differ between daridorexant- and vehicle-treated dogs. The latency to persistent non-REM sleep decreased by 51–69 min after administration of daridorexant versus vehicle reaching 35–53 min. Latency to REM sleep was also decreased from 136 to 50–61 min and was never shorter than the latency to non-REM sleep.

The proportional increase of both non-REM and REM sleep and therewith the preservation of the overall sleep architecture is a unique characteristic of DORAs and differs from that of classical positive GABA-A receptor modulators that skew sleep architecture towards stage N2 of non-REM sleep and a decrease in REM sleep (Bettica et al. 2012; Brisbare-Roch et al. 2007; Brunner et al. 1991; Fox et al. 2013; Lundahl et al. 2012; Tannenbaum et al. 2016). The preclinical data translated clinically as daridorexant improves sleep onset and sleep maintenance in adult and elderly patients with insomnia (Dauvilliers et al. 2020; Zammit et al. 2020).

### Promotion of sleep by daridorexant depends on time of the day, sleep pressure, and orexin levels

Experimental data from our research group support the hypothesis that daridorexant acts most effectively in conditions associated with high orexin neuron activity. This includes situations in which orexin release is ramped up to counteract increasing sleep pressure such as sleep deprivation, insomnia, or the activity phase (Gotter et al. 2013; Modirrousta et al. 2005; Salomon et al. 2003; Tang et al. 2017; Zeitzer et al. 2003). In contrast, daridorexant should have limited effects following a period of rest, such as in the morning following a night of good sleep.

**Fig. 3:**
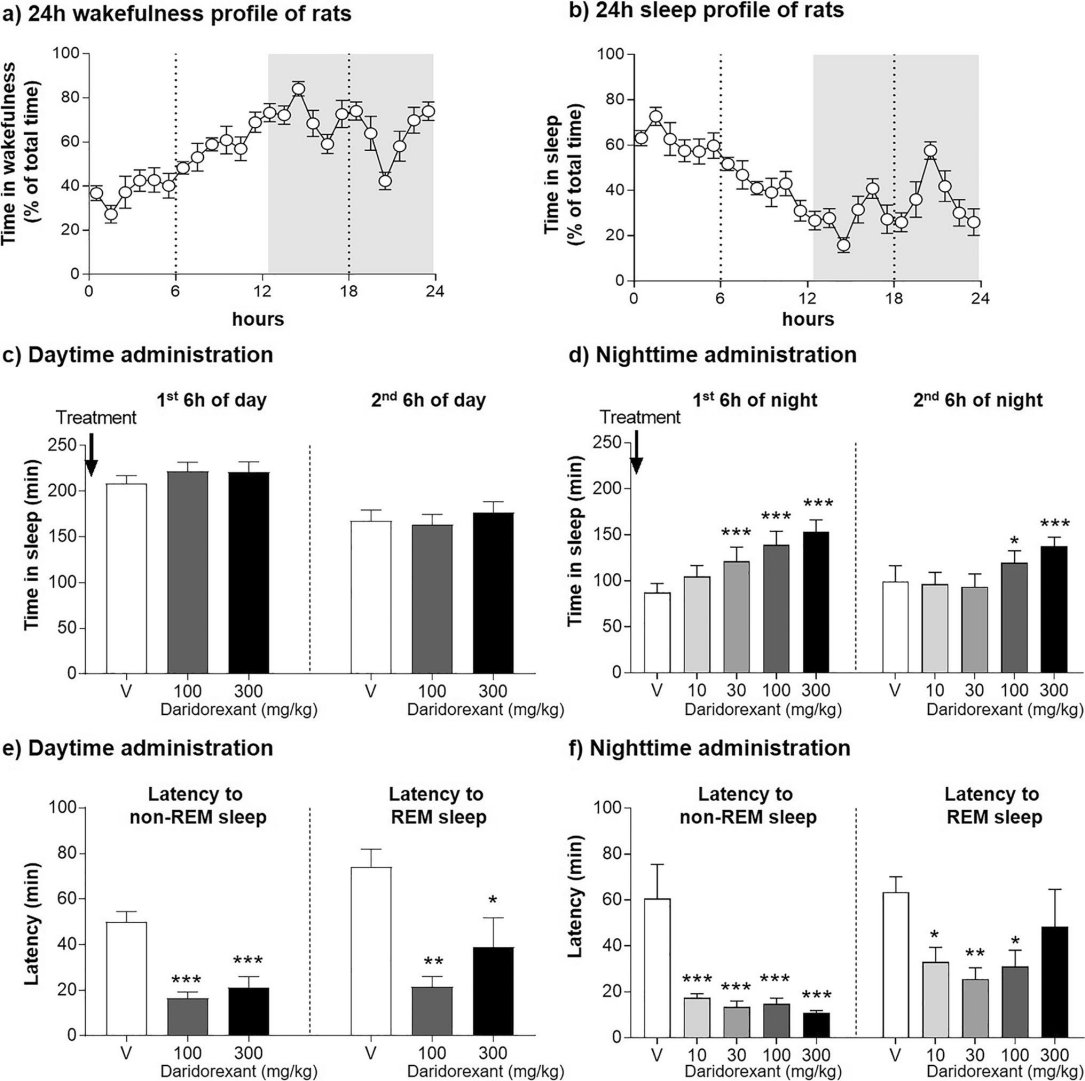
Efficacy of day- or nighttime administration of daridorexant on sleep promotion in rats. Rats are nocturnal animals as illustrated by the wakefulness (**a**) and sleep (**b**) profile over 24 h, depicted in 1 h bins; wakefulness is higher during the 12-h dark phase (gray rectangle). Orexin neurons are active during wakefulness, and highest orexin-A levels in CSF are observed towards the end of the night phase in rats to support wakefulness stabilization when sleep pressure is increasing (Gotter et al. 2013). Orexin-A levels rapidly fall upon sleep. The effect of vehicle (V) or 10, 30, 100, or 300 mg/kg daridorexant on the time spent sleeping (non-REM and REM summarized) following single administration at the beginning of the daytime (**c**) or nighttime (**d**) is summarized by periods of 6 h. Latency to persistent non-rapid eye movement (NREM) sleep (≥ 60 s) and persistent rapid eye movement (REM) sleep (≥ 30 s) was compared to vehicle following daytime (**e**) or nighttime administration (**f**) of daridorexant. Daridorexant was formulated in vehicle (methylcellulose 0.5% in water). Daytime and nighttime administration experiments are two independent crossover studies with *n* = 8 rats each. Data are expressed as mean ± standard error of the mean. One-way ANOVA performed separately for the first and second 6 h of day or night, followed by the post hoc Dunnett’s multiple comparisons test: *p < 0.05, **p < 0.01, ***p < 0.001 vs. vehicle-treated rats. Data-on-file. Further experimental details provided by Boss et al. 2020

Rats are nocturnal animals and spend 60–70% of their dark period awake (Fig. 3a). During their inactive light phase, they spend more time sleeping during the first than during the second half of the phase, with a peak of sleep occurring in the first hours after lights are turned on (Fig. 3b). Administration of daridorexant (up to 300 mg/kg) to rats at the beginning of the light phase (their normal sleep phase) did not increase total time spent asleep (Fig. 3c, data on file) in contrast to the administration at the beginning of the dark (active) phase (Fig. 3d, data-on-file). The evaluation per 6-h period showed the long duration of action of daridorexant at the dose of 100 and 300 mg/kg, the increase of the time spent in sleep still being significant during the second part of the active period (Fig. 3d). In contrast, daridorexant had no effect in the first nor second half of the inactive period (Fig. 3c). The lack of effect of daridorexant on sleep in the first 6 h of the inactive phase can be explained by the already naturally high amount of sleep in this period. It emphasizes that daridorexant primarily works by decreasing wakefulness and that it does not influence sleep per se. The lack of effect of the high and long-acting doses (i.e., 100 and 300 mg/kg) of daridorexant during the second 6 h of the inactive phase can be explained by the limited efficacy of DORAs in conditions of little arousal and low sleep pressure that follow a prior period of sufficient sleep (here, the first 6 h of the inactive period, compared Fig. 3b). The period of time in which natural sleep is less prominent (i.e., the second half of the inactive phase) also corresponds to the one with the lowest reported orexin cerebrospinal fluid (CSF) levels (Gotter et al. 2013), reflecting the reduced level of activity of the orexin neurons during the previous 6-h sleep period.

Daridorexant reduced the latency to persistent non-REM sleep and REM sleep both following administration at the beginning of the inactive (Fig. 3e) and the active phase (Fig. 3f). The likely reason for daridorexant’s effect in the inactive phase is that the procedure of handling and oral gavage supposedly led to an acute increase of orexin neuron firing and OXR activation even during the rats’ normal sleeping phase (note that extracellular orexin levels are highest during periods of heightened emotionality) (Blouin et al. 2013). This activity was counteracted by daridorexant and helped the rats fall asleep faster.

Overall, our data suggest that a residual plasma concentration of daridorexant upon awakening is unlikely to induce next-morning residual effects. This hypothesis is supported by the results of a study from Gotter et al. who treated rhesus monkeys with DORA-22 (another DORA), eszopiclone, or diazepam (Gotter et al. 2013). In this study, despite measurable levels of DORA-22 in the morning following evening treatment, no residual sleep effect and no impairment of memory and attention were observed. In contrast, diazepam generated next-day residual sleep, and both diazepam and eszopiclone induced next-day cognitive deficits.

In agreement with the nonclinical results and its appropriate pharmacokinetic profile (Muehlan et al. 2018), clinical data show that daridorexant is efficacious in promoting sleep in insomniac patients without next-morning residual effects (Dauvilliers et al. 2020; Zammit et al. 2020).

### Surmountable sleep and preservation of motor function

Data from rodents and dogs show that the sleep-promoting effect of daridorexant can be fully and immediately surmounted without causing impairment of motor functions, similar to what is experienced under natural sleep conditions.

**Fig. 4:**
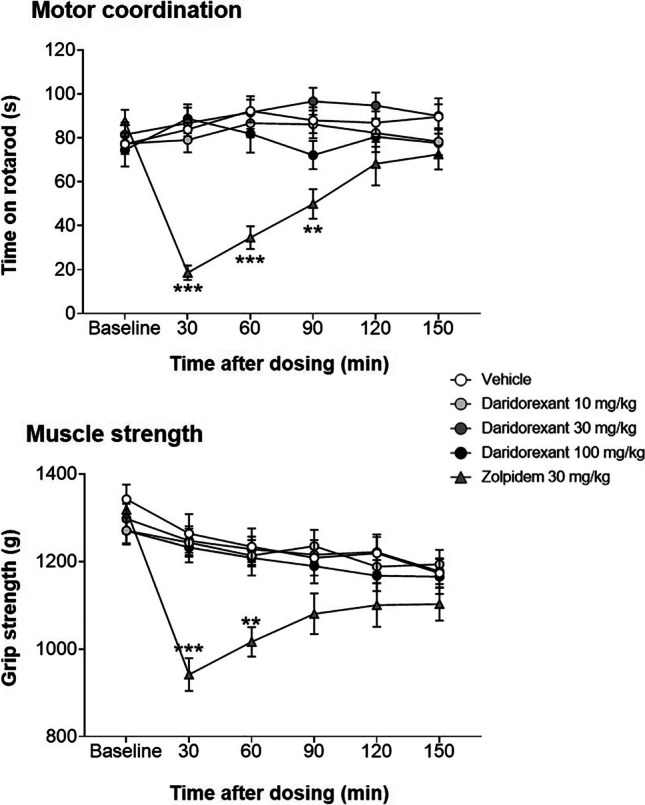
Motor coordination and muscle strength at different time points after single-dose administration of daridorexant in rats and comparison to zolpidem. Effect of a single dose of daridorexant (10, 30, 100 mg/kg), zolpidem (30 mg/kg), and vehicle (0.5% methylcellulose) on motor coordination (rotarod test) and muscle strength (grip strength test) in male Wistar rats. Following drug administration, rats were repeatedly tested at 30, 60, 90, 120, and 150 min post-dose. Baseline performance was measured 60 min before drug administration. Data are means ± standard error of the mean. *N* = 12 rats per dose group. **p < 0.01, ***p < 0.001 vs. vehicle (for each time point), with two-way repeated measure ANOVA followed by Dunnett’s multiple comparisons test. Data-on-file, Idorsia (see Online Resource S2 for experimental details)

The impact of daridorexant on motor coordination and muscle strength was evaluated in rats using the accelerating rotating rod (rotarod) and the forepaw grip strength test (Tang et al. 1995; Voss et al. 2003). Following single-dose oral administration of daridorexant (10, 30, or 100 mg/kg), male Wistar rats were tested repeatedly at different time points (from 30 to 150 min post-dose). All experiments were performed during the light, inactive phase of the day, to mimic the human situation in which patients would take sleep-promoting drugs before bedtime (see Online Resource S2 for experimental details). Daridorexant had no impact on motor coordination (rotarod test) or muscle strength (grip strength test) at any dose or time point studied (Fig. 4, data-on-file). In contrast, when these experiments were performed with zolpidem, given orally at 30 mg/kg (a pharmacologically active dose for sleep induction in rats, compare Fig. 2), performance on both tests was impaired (Fig. 4). These findings suggest that daridorexant does not cause gross or fine motor function impairment in rats and that its sleep-promoting effect can be immediately reversed to full alertness.

**Fig. 5:**
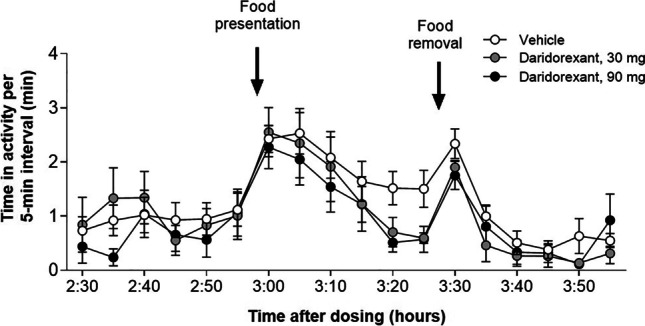
Effect of food presentation on activity following daridorexant administration to dogs. Activity was assessed by automated image quantification using analysis of movements of the dogs (VideoTrack, ViewPoint, France). Food was presented 3 h following oral daridorexant or vehicle administration to male Beagle dogs that were kept under a regular light–dark cycle. Doses are given as total per dog (i.e., a 30 mg/dog dose relates to about 1.7–1.9 mg/kg). The bowl having contained the food was removed 30 min later. Data were integrated over 5-min time intervals and presented as mean ± standard error of the mean of 6–10 dogs per group. Data-on-file; full experimental details provided by Boss et al. 2020

In dogs, sleep-promoting doses of daridorexant did not affect their ability to wake up and behave normally upon presentation of food as a salient positive environmental stimulus (Boss et al. 2020). The dogs’ activity was assessed by automated image quantification using analysis of animal movements (VideoTrack, ViewPoint, France). At the highest doses of daridorexant tested (30 and 90 mg), the dogs awoke instantly and moved and ate normally, upon presentation of food 3 h after drug administration (Fig. 5, data-on-file). Once the food was consumed (within approximately 5 min) and after the bowl having contained the food was removed (30 min later), the dogs went back to sleep. No signs of muscular weakness reminiscent of cataplexy were detected up to 90 mg of daridorexant, based on video recordings as well as electromyography (EMG) and electroencephalogram (EEG) recordings. Cataplexy is a sudden episode of muscle weakness triggered by emotions, without impairment of consciousness, typically observed in narcolepsy type 1 that is caused by orexin neuron loss (Mieda 2017).

The ability to naturally wake up under DORA treatment and react to meaningful stimuli, such as emotionally salient auditory, aversive stimuli, reminiscent of threat, and/or visual stimulation, has also been shown in animals (mice, dogs, and monkeys) treated with almorexant (Brisbare-Roch et al. 2007) or DORA-22 (Brisbare-Roch et al. 2007; Tannenbaum et al. 2014, 2016). Upon awakening, no signs of muscular weakness or cognitive or psychomotor impairment were observed. In addition, once stimulation was stopped, animals rapidly fell back to sleep. In contrast, as reported by Tannenbaum et al. (2016), positive GABA-A receptor modulators abolished the ability of rhesus monkeys to wake up to salient conditioned stimuli, and if they did wake up, their psychomotor performance was impaired (Tannenbaum et al. 2016).

These data support the hypothesis that daridorexant selectively blocks orexin-induced excitation of wake-promoting regions but does not block the possible activation of these regions by other neurotransmitters released when one needs to wake up (Parks et al. 2016). Positive GABA-A receptor modulators, in contrast, induce sleep through widespread inhibition of the CNS, and thus, the threshold for arousal might be increased.

### Preservation of cognition and memory

In addition to the preservation of motor function, nonclinical data suggest that blockade of OX1R and OX2R can promote sleep without impairing cognition and memory (Boss et al. 2014; Dietrich and Jenck 2010; Gamble et al. 2020; Morairty et al. 2014; Uslaner et al. 2013). For example, rats treated with high doses of the DORAs almorexant or ACT-462206 (300 mg/kg) fully retained their spatial reference and working memory, procedural memory, and passive avoidance learning (Boss et al. 2014; Dietrich and Jenck 2010; Morairty et al. 2014).

In addition, cognition has been evaluated in rhesus monkeys following DORA or positive GABA-A receptor modulator administration using match-to-sample performance (a working memory task) and serial choice reaction time accuracy (a measure of attention) (Uslaner et al. 2013). DORA-22 did not disrupt match-to-sample task engagement and did not reduce accuracy in the serial choice reaction time test at any of the doses studied, even at 30-fold greater than the dose that increased sleep (Uslaner et al. 2013). In contrast, monkeys treated with positive GABA-A receptor modulators showed impaired performance in both tasks. These effects occurred at doses below, or similar to, those that promote sleep. These data indicate that DORAs provide a much greater margin between efficacy and potential cognitive disturbances than the currently used positive GABA-A receptor modulators.

This disparity could be explained by neuroanatomical and functional differences of both systems. Even though the orexin receptors are broadly expressed throughout the brain, orexin neurons are restricted to a small area, and orexinergic projections to brain regions other than those involved in regulating sleep and awake states are relatively diffuse (Marcus et al. 2001; Peyron et al. 1998; Sakurai et al. 1998). Activation of additional brain structures by orexins can be regarded as of modulatory nature to coordinate its wakefulness-stabilizing effects with other brain functions (Peyron et al. 1998). In contrast, the GABAergic system is the main inhibitory neurotransmitter in the brain. It controls not only the activity of wake and sleep regulating neurons but also the activity of various other brain centers involved in a multitude of physiological and behavioral functions. Given its ubiquitous role, GABA-A receptors appear to be also heavily expressed in brain regions involved in both attention and memory (amygdala, hippocampus, cerebral cortex), and these regions also receive dense GABAergic innervation (Akbarian et al. 1995; Fritschy and Mohler 1995).

In a clinical study with healthy young adults, almorexant did not impair cognitive performance in complex tasks of verbal memory or executive function (Neylan et al. 2020). A single dose of zolpidem or almorexant produced similar levels of subjective sleepiness and impairment regarding the ability to maintain wakefulness in a dark, low-stimulus environment in which the subject’s only task was to remain awake. However, for more complex tasks of verbal memory or executive function, performance was not impaired with almorexant, but was significantly impaired with zolpidem. The authors hypothesize that the sleep-promoting effect of DORAs is permissive for wake-promoting systems to be recruited in the setting of a task demand. It should, however, be noted that dosing was in the afternoon and performance following awakening from sleep was not tested.

Overall, these nonclinical and clinical observations support the hypothesis that DORAs promote sleep without the cognitive side effects (i.e., impairment of attention and memory) that are often seen with the positive GABA-A receptor modulators commonly used as insomnia treatments (Berlin et al. 1993; Kleykamp et al. 2012; Morairty et al. 2014; Roehrs et al. 1994; Uslaner et al. 2013). Data from the daridorexant phase 3 program, using the Insomnia Daytime Symptoms and Impacts Questionnaire (IDSIQ), a validated patient-reported outcome instrument (Hudgens et al. 2020), showed that daridorexant improved patients’ daytime performance.

### Efficacy without tolerance or rebound

Insomnia is a chronic disorder. Yet development of tolerance (decrease in efficacy with time) and rebound insomnia (necessitating slow down-titration upon interruption of long-term treatment) are often seen with traditional hypnotics (Kripke 2000; Wilson et al. 2019; Zammit 2009). Nonclinical data indicate that daridorexant does not cause tolerance or risk of rebound.

**Fig. 6:**
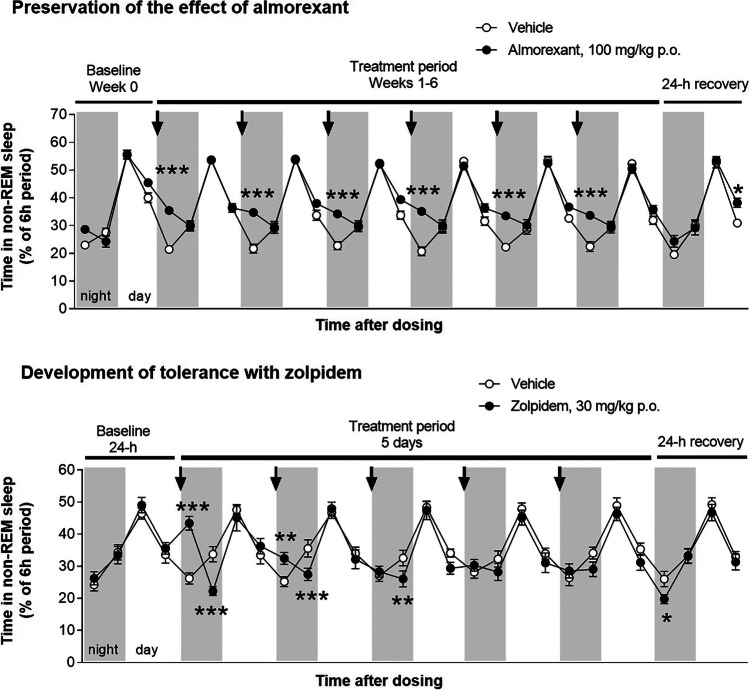
Effect of daily, repeated administrations of almorexant and zolpidem on sleep promotion in rats. Male Wistar rats received daily repeated oral administrations of almorexant (100 mg/kg p.o.) or vehicle at the beginning of the night (arrows), active period (gray rectangles) for 6 weeks, followed by 7 days of recovery. Sleep was recorded via EEG/EMG-based telemetry. Data were averaged across several days per week, except for the first recovery night and day; 2 days/nights for week 0, and 4 days/nights for each of weeks 1–6. Repeated administration was also performed with zolpidem (30 mg/kg p.o.) for 5 consecutive days, followed by 1 day of recovery. Almorexant data are represented as the mean ± standard error of the mean of eight rats per group. Zolpidem data are presented as the mean ± standard error of the mean of seven rats per group. Two-way ANOVA followed by post hoc Bonferroni test: *p < 0.05, **p < 0.01, ***p < 0.001, vs. the vehicle-treated group. REM, rapid eye movement; EEG, electroencephalography; EMG, electromyography. Data-on-file (see Online Resource S3 for experimental details)

Rodent data using DORAs consistently suggest that the sleep-promoting efficacy of these compounds is sustained upon extended treatment without evidence of rebound (Beuckmann et al. 2019; Brisbare-Roch et al. 2008, 2007, 2010). Almorexant was administered daily for 42 days (100 mg/kg/day p.o. at the beginning of the active phase) in rats to assess its long-term effect on sleep by EEG/EMG (data-on-file; see Online Resource S3 for experimental details). The sleep-promoting effect of almorexant was maintained for the entire duration of treatment (Fig. 6). Almorexant repeatedly increased the time spent in both non-REM (Fig. 6) and REM sleep without altering the proportion of non-REM and REM sleep over the total sleep time (non-REM sleep varied from 81 to 84% of total sleep time under vehicle treatment, and from 82 to 84% under almorexant treatment). No signs of tolerance were observed (data-on-file). The sleep-promoting effect of almorexant was mainly evident during the first half of the nights following administration without compensation by increased wakefulness during the second part of the nights (or during the following days). Abrupt discontinuation after 42 days of treatment did not lead to any wakefulness rebound during the first 24 h, and animals returned to their normal sleep/wake cycle, comparable to that of the vehicle-treated animals (Fig. 6; data-on-file). Tachyphylaxis caused by receptor downregulation is not expected with orexin receptor antagonism in contrast to what is observed with GABA-A receptor agonists (Svob Strac et al. 2008). Indeed, daily repeated administration of zolpidem in rats for only 5 consecutive days (30 mg/kg p.o.), when administered at the beginning of the active phase, induced tolerance after already the second administration (Fig. 6; data-on-file).

The increase of the time spent asleep during the first 6-h period of the night completely faded upon repeated treatment with zolpidem. The magnitude of the effect on non-REM sleep (the most affected variable) already decreased after the second administration and was no longer significant by the third administration. In addition, the increase of non-REM sleep observed in the first 6-h period after zolpidem administration was followed by an immediate compensation characterized by a significant decrease of non-REM sleep time during the second 6-h period of the night. Moreover, during the first 24 h after treatment discontinuation, sleep was slightly reduced during the first 6-h period of the night, indicating further wakefulness rebound (Fig. 6; data-on-file).

Clinical data up to 12 months with suvorexant have shown a consistent sleep-promoting effect over time (Michelson et al. 2014). Phase 2 clinical data with daridorexant support the lack of tolerance development to chronic treatment; efficacy of daridorexant was maintained up to 1 month in patients with insomnia, with no signals suggestive of withdrawal syndrome or rebound insomnia following cessation of daridorexant (Dauvilliers et al. 2020). In the phase 3 studies, the 3-month duration of double-blind treatment allows to explore longer-term effects of daridorexant in addition to further assessing the potential for rebound and withdrawal.

### Lack of abuse potential

Abuse is a concern for sleep medications. CNS-depressant drugs belonging to the class of benzodiazepines as well as Z-drugs have been widely prescribed for their sleep-inducing effects. Both drug classes are known to be abused and to cause dependence in humans (Schifano et al. 2019). Pharmaco-epidemiological studies across the UK, France, Germany, and the US have confirmed that Z-drugs are abused to a significant extent by both patients and recreational drug users, although generally less often than benzodiazepines (Davies et al. 2017; Hoffmann and Glaeske 2014; Lee et al. 2014; Rousselet et al. 2017).

In support of the clinical picture, in animals, zopiclone, zaleplon, and zolpidem have shown reinforcing effects, as well as withdrawal symptoms upon discontinuation after chronic use, indicative of abuse and dependence potential for humans (Ator 2000; Ator et al. 2000; Griffiths et al. 1992; Yanagita 1982). For instance, in baboons, trained to self-administer cocaine, zolpidem was readily self-administered upon substitution (Griffiths et al. 1992). Zolpidem also produced withdrawal symptoms of medium severity in baboons upon treatment discontinuation after repeated daily administrations (Weerts et al. 1998). Moreover, Z-drugs generalize to benzodiazepines in drug-discrimination studies, indicating that the interoceptive effects perceived by animals are similar to those of benzodiazepines (e.g., Griffiths et al. 1992).

In contrast to those data generated in nonclinical studies with Z-drugs, DORAs have so far not revealed any signs in animals that are indicative of abuse potential in man. For example, in a conditioned place preference (CPP) experiment, in which rats learn to associate a compartment of a testing chamber to the rewarding properties of a drug, rats did not display any preference for the almorexant-paired compartment (Steiner et al. 2013b). In contrast, ɣ hydroxybutyrate (GHB), a sleep-inducing drug acting through the GABA-B receptor, caused a significant place preference in the same setup (Steiner et al. 2013b). This is consistent with the reported abuse liability of GHB in patients (Griffiths and Johnson 2005).

During the CPP conditioning procedure, both almorexant and GHB reduced locomotion upon first administration, as expected for sleep-promoting agents. While hypolocomotion was maintained upon repeated administration with almorexant, rats developed tolerance to the hypolocomotor effects of GHB from the third administration onwards (Steiner et al. 2013b). This supports the notion that medications that induce tolerance (exemplified by GHB) are more likely to cause dependence and exert abuse potential than those that do not (such as DORAs).

The abuse potential of daridorexant was tested extensively in a nonclinical setting and a high-level overview has been published as a poster at the American Congress of Neuropsychopharmacology (Ufer et al. 2020). In short, daridorexant did not bind to any known abuse-associated CNS targets at clinically relevant concentrations, based on molecular profiling (compare Supplementary file S1). Daridorexant (up to 1 mg/kg per intravenous infusion) did not elicit self-administration in rats suggesting a lack of reinforcing effects. Daridorexant’s potential interoceptive effects (up to 60 mg/kg p.o.) did not generalize to those produced by zolpidem (3 mg/kg p.o.) in a drug discrimination rat study. Finally, rats treated chronically with daridorexant (up to 200 mg/kg p.o. per day) did not develop any withdrawal signs upon treatment discontinuation indicating lack of physical dependence.

The abuse of sleep medications is a major concern to both prescribing physicians and patients (Griffiths and Johnson 2005). Extreme caution is needed when considering their prescription to patients with a history of abuse or dependence, or with psychiatric comorbidities (Hajak et al. 2003). Daridorexant is devoid of any abuse potential or dependence signals in animals. In agreement with our findings, daridorexant also did not cause any withdrawal symptoms after treatment discontinuation in its phase 2 clinical trial (Dauvilliers et al. 2020).

### Beyond the sleep effect: the potential for improvement of daytime functioning

The pathophysiology of insomnia is unclear; however, one model (the cognitive model) suggests that insomnia can be triggered by a stressful life event which is ruminated on and leads to an acute episode of insomnia. Thereafter the worries and ruminations about life stresses shift to worries about insomnia itself and the associated daytime consequences (Buysse et al. 2011; Levenson et al. 2015; Roth 2007). Insomnia is indeed associated with significant distress or impairment in daytime functioning including fatigue, daytime sleepiness, mood disturbances, reduced cognitive function, performance, and motivation, as well as behavioral problems (American Academy of Sleep Medicine 2014; Wilson et al. 2019). The goal of developing daridorexant for the treatment of insomnia was to improve the nighttime symptoms but, ideally, also the daytime symptoms experienced by patients. This was the rationale for developing the patient-reported outcome tool IDSIQ and using it to assess daytime function in the phase 3 clinical program (Hudgens et al. 2020).

The equipotency of daridorexant on the OX1R and the OX2R may be an asset in the treatment of insomnia. Although the repeated improvement of sleep, night after night, can reduce the fear of not being able to sleep properly, inhibition of both OX1R and OX2R may have additional benefit over inhibition of only OX2R which has been shown to be sufficient to promote sleep in human (De Boer et al. 2018). Indeed, selective OX1R antagonists have been shown to reduce fear-related behavior (Soya and Sakurai 2020b; Steiner et al. 2013a) which suggests that daridorexant, via OX1R antagonism, could contribute to the reduction of anxiety and stress per se, in addition to the reduction of wakefulness.

Preclinical data with daridorexant and other DORAs support the concept that anxiety, mood alteration, sympathetic hyperactivation, and cognitive impairment, developed as a result of insomnia, could be improved with daridorexant treatment.

Studies in rats showed that daridorexant (10, 30, and 100 mg/kg) exerts a dose-dependent anxiolytic-like effect in three different models assessing some aspects of human anxiety (Steiner et al. 2020): the fear-potentiated startle test (modeling anxiety states reminiscent of post-traumatic stress disorder), the social stress–induced hyperthermia test (modeling aspects of social anxiety disorder), and the schedule-induced polydipsia test (modeling the compulsive stress component of obsessive–compulsive disorder). This pharmacological profile is consistent with that of other DORAs, including almorexant and ACT-462206, which have shown anxiolytic-like effects at sleep-promoting doses in similar models (Boss et al. 2014; Steiner et al. 2012; Viviani et al. 2015).

Almorexant is also known to exert antidepressant-like effects in mice (Nollet et al. 2011, 2012), to reduce sympathetic hyperactivity in rats (Furlong et al. 2009; Li et al. 2013), and to allow rats to fall asleep faster after experiencing a stressful situation (Steiner et al. 2013c).

Interestingly, a recent study (2020) showed that, in a rat model of mild stress-induced insomnia, treatment with DORA-22 improved the memory deficits associated with the disease (Gamble et al. 2020). In this model (consisting of double-dirty cage changes), an alteration of the sleep/wake profile, evocative of insomnia, is induced when rats are exposed to a cage dirtied by another unfamiliar rat for 3 h and then another dirty cage 3 h later (McKenna et al. 2019). This procedure induced deficits in spatial memory recall performance in the Morris water maze test. DORA-22 improved the memory impairment associated with the model (Gamble et al. 2020). One possible explanation was that DORA-22 treatment was associated with an increase in the number and average duration of non-REM sleep spindles within the first hour of treatment. Sleep spindles are proposed to play a role in memory consolidation (Fogel and Smith 2011; Manoach and Stickgold 2019) and thus may have contributed to the improved memory that was observed.

Based on these preclinical results, daridorexant could possibly reduce the worry of not being able to sleep and thus perform poorly during the day, and at the same time improve the impairments in daytime function, including anxiety symptoms and memory issues, that can be a direct consequence of the insomnia. The IDSIQ used in the clinical phase 3 trials of daridorexant (Hudgens et al. 2020) showed that daridorexant improved patients’ daytime performance.

## Future perspectives

Insomnia is, either as a cause or consequence, often associated with other diseases, including psychiatric disorders and cardiovascular or neurodegenerative diseases (Wilson et al. 2019). Treatment of insomnia with daridorexant, by improving sleep and daytime functioning, has the potential to also provide benefits to those associated comorbidities.

Among psychiatric diseases, patients suffering from opioid use disorder frequently experience symptoms of insomnia and anxiety that are correlated to their drug craving and are considered significant contributors to relapse during periods of abstinence (Ferri et al. 2014; Frers et al. 2021; Luca and Peris 2020; Teeters et al. 2020). The same holds true for other types of substance use disorders including ketamine (Yen et al. 2020), cocaine (DiGirolamo et al. 2017), and alcohol use disorder (Conroy and Arnedt 2014). The combined effectiveness of daridorexant in exerting sleep-promoting and anxiolytic-like effects in animals could make it an ideal drug candidate for investigating its usefulness as complementary symptomatic treatment for patients suffering from substance use disorder wanting to become or remain abstinent.

Insomnia, a disorder of hyperarousal, is associated with chronic sympathetic hyperactivity (Buysse et al. 2011; Levenson et al. 2015; Riemann et al. 2010; Roth et al. 2007) and increases the risk of hypertension and cardiovascular diseases (Wilson et al. 2019). Upregulation of orexin signaling might contribute to the development of hypertension (Huber et al. 2017; Li et al. 2013). Given that DORAs reduce sympathetic drive and blood pressure in animals (Furlong et al. 2009; Li et al. 2013), treatment of insomnia with daridorexant could have additional therapeutic potential in patients with hypertension and/or at risk of developing cardiovascular diseases.

The prevalence of insomnia increases with age and can become a risk factor for developing dementia (Jelicic et al. 2002; Potvin et al. 2012). Several studies highlight a bidirectional interaction between sleep disturbances and Alzheimer’s disease (AD) with sleep disturbances leading to increased AD pathology which, in turn, exacerbates the sleep problems (Kang et al. 2017). Positive GABA-A modulators impair memory and cognition and are associated with higher risk of dementia or AD. They are not recommended for the treatment of comorbid insomnia in AD (He et al. 2019; Tapiainen et al. 2018). In contrast, daridorexant does not impair learning and memory and could have beneficial effects in the prevention of dementia. Almorexant was able to slow down disease progression in a mouse model of AD (Kang et al. 2009).

## Conclusions

Current pharmacological options for the treatment of chronic insomnia do not meet the needs for all patients. Targeting the orexin system represents a promising therapeutic option. Daridorexant, a DORA, was discovered, thanks to an intense drug discovery program aimed at optimizing both the efficacy and pharmacokinetic profile of a sleep-promoting agent. Animal data show that daridorexant can effectively promote sleep and maintain a natural sleep architecture, without impairing the ability to arouse in response to salient stimuli and without impairing motor function. Consistent with other DORAs, daridorexant is also expected to preserve cognitive function, to have very low abuse potential, and to not induce tolerance or rebound following chronic use, thus overcoming many of the limitations associated with more traditional hypnotic medications. A comprehensive clinical pharmacology program has been conducted in parallel to the phase 3 trials, and it is hoped that the presented promising nonclinical data of daridorexant will translate to humans.

## Supplementary Information

Below is the link to the electronic supplementary material.

Supplementary file1 (DOCX 52.6 KB)
