# Supplementary material for: Nonclinical pharmacology of daridorexant: a new dual orexin receptor antagonist for the treatment of insomnia
Source: Psychopharmacology (Berl). 2021 Aug 20;238(10):2693–708. doi: 10.1007/s00213-021-05954-0 (PMC8455402; doi:10.1007/s00213-021-05954-0)
Supplement: Supplementary file 1 — Supplementary file1 (DOCX 52.6 KB) [file 213_2021_5954_MOESM1_ESM.docx]

**SUPPLEMENTARY MATERIAL**

**Nonclinical pharmacology of daridorexant: a new dual orexin receptor antagonist for the treatment of insomnia**

**Authors:** Catherine Roch^1^, Giorgio Bergamini^1^, Michel Steiner^1^, Martine Clozel^1^

**Affiliation: ^1^**Idorsia Pharmaceuticals Ltd, Allschwil, Switzerland.

**Corresponding author:**

Catherine Roch

Idorsia Pharmaceuticals Ltd, Allschwil, Switzerland

Email: [catherine.roch@idorsia.com](mailto:catherine.roch@idorsia.com)

Telephone: +41 58 844 01 69

**S1. Panel screen of pharmacological targets in the evaluation of compound ACT-541468 (daridorexant)**

Biochemical assay results are presented as the percent inhibition of specific binding or activity.

| **Assay name** | **Species** | **Rep.** | **Conc.** | **% Inhibition** |
| --- | --- | --- | --- | --- |
| Cholinesterase, Acetyl, ACES | Human | 2 | 10 µM | 8 |
| Cyclooxygenase COX-1 | Human | 2 | 10 µM | 2 |
| Cyclooxygenase COX-2 | Human | 2 | 10 µM | 5 |
| Lipase | Human | 2 | 10 µM | 1 |
| Monoamine Oxidase MAO-A | Human | 2 | 10 µM | 8 |
| Monoamine Oxidase MAO-B | Human | 2 | 10 µM | 7 |
| Peptidase, CTSL (Cathepsin L) | Human | 2 | 10 µM | -14 |
| Phosphodiesterase PDE1 | Bovine | 2 | 10 µM | 20 |
| Phosphodiesterase PDE10A2 | Human | 2 | 10 µM | 7 |
| Phosphodiesterase PDE2 | Human | 2 | 10 µM | 9 |
| Phosphodiesterase PDE3 | Human | 2 | 10 µM | 5 |
| Phosphodiesterase PDE4 | Human | 2 | 10 µM | 81 |
| Phosphodiesterase PDE5 | Human | 2 | 10 µM | 21 |
| Phosphodiesterase PDE6 | Bovine | 2 | 10 µM | 2 |
| Steroid 5α-Reductase | Rat | 2 | 10 µM | 11 |
| Adenosine A_1_ | Human | 2 | 10 µM | 10 |
| Adenosine A_2A_ | Human | 2 | 10 µM | 21 |
| Adenosine A_2B_ | Human | 2 | 10 µM | 17 |
| Adenosine A_3_ | Human | 2 | 10 µM | 17 |
| Adrenergic α_1A_ | Rat | 2 | 10 µM | 13 |
| Adrenergic α_1B_ | Rat | 2 | 10 µM | -8 |
| Adrenergic α_1D_ | Human | 2 | 10 µM | -7 |
| Adrenergic α_2A_ | Human | 2 | 10 µM | 0 |
| Adrenergic α_2B_ | Human | 2 | 10 µM | 10 |
| Adrenergic α_2C_ | Human | 2 | 10 µM | 0 |
| Adrenergic β_1_ | Human | 2 | 10 µM | 7 |
| Adrenergic β_2_ | Human | 2 | 10 µM | 3 |
| Adrenergic β_3_ | Human | 2 | 10 µM | 49 |
| Aldosterone | Rat | 2 | 10 µM | -4 |
| Angiotensin AT_1_ | Human | 2 | 10 µM | 22 |
| Angiotensin AT_2_ | Human | 2 | 10 µM | 1 |
| Bombesin, Non-Selective | Rat | 2 | 10 µM | 20 |
| Calcium Channel L-Type, Benzothiazepine | Rat | 2 | 10 µM | 8 |
| Calcium Channel L-Type, Dihydropyridine | Rat | 2 | 10 µM | -11 |
| Calcium Channel L-Type, Phenylalkylamine | Rat | 2 | 10 µM | 48 |
| Calcium Channel N-Type | Rat | 2 | 10 µM | 17 |
| Cannabinoid CB_1_ | Human | 2 | 10 µM | 15 |
| Cannabinoid CB_2_ | Human | 2 | 10 µM | 8 |
| Cholecystokinin CCK_1_ (CCK_A_) | Human | 2 | 10 µM | 18 |
| Cholecystokinin CCK_2_ (CCK_B_) | Human | 2 | 10 µM | 0 |
| Corticotropin Releasing Factor CRF_1_ | Human | 2 | 10 µM | 5 |
| Dopamine D_1_ | Human | 2 | 10 µM | -3 |
| Dopamine D_2L_ | Human | 2 | 10 µM | 5 |
| Dopamine D_2S_ | Human | 2 | 10 µM | -1 |
| Dopamine D_3_ | Human | 2 | 10 µM | 9 |
| Dopamine D_4.2_ | Human | 2 | 10 µM | -1 |
| Dopamine D_4.4_ | Human | 2 | 10 µM | 11 |
| Dopamine D_4.7_ | Human | 2 | 10 µM | 3 |
| Dopamine D_5_ | Human | 2 | 10 µM | -8 |
| Endothelin ET_A_ | Human | 2 | 10 µM | -2 |
| Endothelin ET_B_ | Human | 2 | 10 µM | 2 |
| Epidermal Growth Factor (EGF) | Human | 2 | 10 µM | 0 |
| Estrogen ERα | Human | 2 | 10 µM | 14 |
| Estrogen ERβ | Human | 2 | 10 µM | 3 |
| GABA_A_, Chloride Channel, TBOB | Rat | 2 | 10 µM | 18 |
| GABA_A_, Chloride Channel, TBPS | Rat | 2 | 10 µM | 27 |
| GABA_A_, Flunitrazepam, Central | Rat | 2 | 10 µM | 7 |
| GABA_A_, Muscimol, Central | Rat | 2 | 10 µM | 2 |
| GABA_B1A_ | Human | 2 | 10 µM | 10 |
| GABA_B1B_ | Human | 2 | 10 µM | 9 |
| Galanin GAL1 | Human | 2 | 10 µM | -6 |
| Galanin GAL2 | Human | 2 | 10 µM | 6 |
| γ-Hydroxybutyric Acid (GHB) Receptor | Rat | 2 | 10 µM | 10 |
| Glucocorticoid | Human | 2 | 10 µM | 15 |
| Glutamate, AMPA | Rat | 2 | 10 µM | 0 |
| Glutamate, Kainate | Rat | 2 | 10 µM | 3 |
| Glutamate, NMDA, Glycine | Rat | 2 | 10 µM | -6 |
| Glutamate, NMDA, Phencyclidine | Rat | 2 | 10 µM | 2 |
| Glutamate, Non-Selective | Rat | 2 | 10 µM | 12 |
| Growth Hormone Secretagogue (GHS, Ghrelin) | Human | 2 | 10 µM | 1 |
| Histamine H1 | Human | 2 | 10 µM | -14 |
| Histamine H2 | Human | 2 | 10 µM | 1 |
| Histamine H3 | Human | 2 | 10 µM | 0 |
| Histamine H4 | Human | 2 | 10 µM | 16 |
| Leptin | Mouse | 2 | 10 µM | 5 |
| Melanocortin MC1 | Human | 2 | 10 µM | 0 |
| Melanocortin MC3 | Human | 2 | 10 µM | 1 |
| Melanocortin MC4 | Human | 2 | 10 µM | 5 |
| Melanocortin MC5 | Human | 2 | 10 µM | -3 |
| Melatonin MT1 | Human | 2 | 10 µM | 7 |
| Melatonin MT2 | Human | 2 | 10 µM | 38 |
| Muscarinic M1 | Human | 2 | 10 µM | 30 |
| Muscarinic M2 | Human | 2 | 10 µM | 11 |
| Muscarinic M3 | Human | 2 | 10 µM | 11 |
| Muscarinic M4 | Human | 2 | 10 µM | 4 |
| Muscarinic M5 | Human | 2 | 10 µM | 7 |
| Neuromedin U NMU1 | Human | 2 | 10 µM | 12 |
| Neuromedin U NMU2 | Human | 2 | 10 µM | 7 |
| Neuropeptide Y Y1 | Human | 2 | 10 µM | 0 |
| Neuropeptide Y Y2 | Human | 2 | 10 µM | 3 |
| Neurotensin, Non-Selective | Mouse | 2 | 10 µM | 1 |
| Nicotinic Acetylcholine | Human | 2 | 10 µM | -16 |
| Opiate δ1 (OP1, DOP) | Human | 2 | 10 µM | -5 |
| Opiate κ(OP2, KOP) | Human | 2 | 10 µM | 6 |
| Opiate µ(OP3, MOP) | Human | 2 | 10 µM | 8 |
| Orphanin ORL1 | Human | 2 | 10 µM | 1 |
| Oxytocin | Human | 2 | 10 µM | 34 |
| Potassium Channel [KA] | Rat | 2 | 10 µM | 4 |
| Potassium Channel [KATP] | Hamster | 2 | 10 µM | 7 |
| Potassium Channel [SKCA] | Rat | 2 | 10 µM | 4 |
| Potassium Channel hERG | Human | 2 | 10 µM | 21 |
| Purinergic P2X | Rabbit | 2 | 10 µM | 0 |
| Purinergic P2Y | Rat | 2 | 10 µM | 0 |
| Serotonin (5-Hydroxytryptamine) 5-HT1, Non-Selective | Rat | 2 | 10 µM | 33 |
| Serotonin (5-Hydroxytryptamine) 5-HT1A | Human | 2 | 10 µM | 2 |
| Serotonin (5-Hydroxytryptamine) 5-HT1B | Rat | 2 | 10 µM | -3 |
| Serotonin (5-Hydroxytryptamine) 5-HT1B | Human | 2 | 10 µM | -6 |
| Serotonin (5-Hydroxytryptamine) 5-HT2, Non-Selective | Rat | 2 | 10 µM | 8 |
| Serotonin (5-Hydroxytryptamine) 5-HT2A | Human | 2 | 10 µM | -3 |
| Serotonin (5-Hydroxytryptamine) 5-HT2B | Human | 2 | 10 µM | 9 |
| Serotonin (5-Hydroxytryptamine) 5-HT2C | Human | 2 | 10 µM | -10 |
| Serotonin (5-Hydroxytryptamine) 5-HT3 | Human | 2 | 10 µM | 6 |
| Serotonin (5-Hydroxytryptamine) 5-HT4 | Guinea pig | 2 | 10 µM | 34 |
| Serotonin (5-Hydroxytryptamine) 5-HT5A | Human | 2 | 10 µM | 5 |
| Serotonin (5-Hydroxytryptamine) 5-HT6 | Human | 2 | 10 µM | 1 |
| Serotonin (5-Hydroxytryptamine) 5-HT7 | Human | 2 | 10 µM | -3 |
| Sigma σ1 | Human | 2 | 10 µM | -10 |
| Sigma σ2 | Rat | 2 | 10 µM | 11 |
| Somatostatin sst1 | Human | 2 | 10 µM | 14 |
| Somatostatin sst2 | Human | 2 | 10 µM | -3 |
| Somatostatin sst3 | Human | 2 | 10 µM | 4 |
| Somatostatin sst4 | Human | 2 | 10 µM | 4 |
| Somatostatin sst5 | Human | 2 | 10 µM | -1 |
| Tachykinin NK1 | Human | 2 | 10 µM | -2 |
| Tachykinin NK2 | Human | 2 | 10 µM | 7 |
| Tachykinin NK3 | Human | 2 | 10 µM | 23 |
| Transporter, Dopamine (DAT) | Human | 2 | 10 µM | 52 |
| Transporter, GABA | Rat | 2 | 10 µM | -10 |
| Transporter, Norepinephrine (NET) | Human | 2 | 10 µM | 17 |
| Transporter, Serotonin  (5-Hydroxytryptamine) (SERT) | Human | 2 | 10 µM | -11 |
| Vasopressin V1A | Human | 2 | 10 µM | -18 |
| Vasopressin V1B | Human | 2 | 10 µM | 5 |
| Vasopressin V2 | Human | 2 | 10 µM | 5 |

Items meeting criteria for significance (≥50% stimulation or inhibition) are highlighted

**S2: Effect of single-dose oral administration of daridorexant (ACT-541468) on motor functions in male Wistar rats**

**Methods**

**Animals**

All experimental procedures were approved by the local Veterinary Office and adhered to Swiss federal regulations on animal experimentation. Male Wistar Han rats (Charles River; Sulzfeld, Germany) were maintained under standard laboratory conditions (temperature 20– 21ºC, relative humidity 55–70%, and food and water *ad libitum*) under a regular 12-h light-dark cycle (lights on at 06:00 hours). All experiments were performed during the light phase, which is the inactive phase of the rats, between 08:00 and 18:00 hours. On the test day, rats were 10–12 weeks old, and their body weight was 304–410 g.

**Drugs and formulations**

The hydrochloride salt (ACT-541468A) of daridorexant (ACT-541468) was formulated in an aqueous solution of 0.5 % methyl cellulose (MC) for oral gavage at 10, 30, and 100 mg/5 mL/kg (doses were calculated for the free base). Zolpidem was formulated in an aqueous solution of MC 0.5 % for oral gavage at 30 mg/5 mL/kg.

**Rotarod test**

The rotarod was a fully automated equipment from Omnitech electronics (Columbus, Ohio, USA) controlled by a PC with the software Accurotor (v6.1, Fusion). The rotarod consisted of a motor-driven apparatus with a rotating cylinder (rod), located above a platform and is a measure of motor coordination and gross motor skills. Once rats fell off the rod, a digital timer controlled by the platform automatically recorded the time spent before on the rotating rod. Two rats were tested in parallel at a time on the same rotarod side-by-side, separated by a plastic divider.

*Habituation*. Rats were first habituated to general handling by the experimenter for 2 days (approximately 2 min each time). Rats were then habituated to the rotarod apparatus: they were initially placed once on the rotarod at constant speed (5 rpm for 2 min), for habituation to the equipment and then exposed once to a variable speed (0 to 20 rpm in 1 min).

*Rotarod training*. On the training day, rats were placed on the rotarod with an acceleration schedule of 0 to 40 rpm in 2 min; this was repeated three times in the morning from 9:30 to 12:30 (with 30 min in-between sessions) and three times in the afternoon from 14:00 to 17:30 (with 30 min in-between sessions). The test was performed the following day.

*Rotarod test*. On the test day, rats received one additional ‘reminder’ session using the same acceleration schedule as during the training (0 to 40 rpm in 2 min). The individual baseline rotarod performance was calculated by averaging the time spent on the rotarod during the last training trial on the day before testing and the time spent on the rotarod during the single ‘reminder’ training trial on the test day. Baseline performance was used to allocate and evenly distribute rats with similar overall performance to the different treatment groups. Rats were administered with drugs 60 min after the ‘reminder’ baseline trial on the test day, and were then repeatedly tested on the rotarod (0 to 40 rpm in 2 min) at 30, 60, 90, 120, and 150 min after administration to provide a profile of the drug effect over time.

**Grip test**

The grip strength test used a triangular pull bar (2 mm in diameter and 5 cm wide) connected to a digital strain gauge (BIOSEB, Vitrolles, France) to measure changes in the forelimb grip strength of rats. Animals were held by the tail. After grasping the bar with the front paws (test of fine motor skills), they were gently pulled away from the bar in a smooth, steady motion, until they released the bar. The strain gauge measured the force (g) required to break the rat’s grip. For each measurement, three readings were taken for each rat (with 10 seconds of rest in the home cage in-between). The average force of these three readings was used as the individual grip strength score of each rat. The individual baseline grip strength performance was measured immediately after the single “reminder” rotarod session before drug treatment. Then, after drug administration, rats were repeatedly tested immediately after each rotarod test (at 30, 60, 90, 120, and 150 min).

**Experimental settings**

For practicality reasons, the 60 rats (n=12 allocated to each treatment group) enrolled into the experiment were tested at different days (n=15 rats / day; representing each time 3 rats of each treatment group).

**Statistical analysis**

Data are presented as mean ± SEM. One-way ANOVA was used to analyze baseline performance on rotarod and baseline grip strength. Statistical analysis of drug effects on rotarod performance and grip strength were performed using two-way ANOVA with “Treatment” as independent factor and “Time” as dependent, repeated measure, factor, followed by Dunnett’s multiple comparisons test.

**S3. Effect of 6 weeks daily oral administrations of almorexant and 5 days daily administration of zolpidem on the sleep-wake cycle of normal Wistar rats**

**Methods**

**Animals**

All experimental procedures were approved based on international guidelines and adherence to Swiss federal regulations on animal experimentation. Male Wistar rats (RCC Ltd) were maintained under standard laboratory conditions (temperature 20 ± 2°C, relative humidity 55–70% and food and water ad libitum) under a 12 h light-dark cycle.

**Formulation and administration**

Almorexant (hydrochloride salt) was formulated in an aqueous solution of MC 0.25% for oral gavage at 100 mg/5 ml/kg. The drug-treated group was counterbalanced with a vehicle-treated control group (8 animals per group). Administrations were performed at the beginning of the night every day for 42 days.

Zolpidem was formulated in an aqueous solution of MC 0.25% for oral gavage at 30 mg/5 mL/kg. The experiment followed a crossover design with 5 consecutive days of vehicle or drug administration separated by 9 days of wash-out (7 animals).

**Radiotelemetry and implantation**

EEG, EMG, and home cage activity were measured by telemetry as described previously (Boss et al. 2020) using TL11M2-F20-EET implants (Data Science International, St Paul, MN, USA). The entire surgical implantation was performed as described previously (Boss et al. 2020) under general anesthesia with an intra-peritoneal injection of a mixture of ketamin-HCl and xylazin. Following at least 2 weeks of recovery from surgery, animals, in their home cage, were divided in two groups (vehicle and treated group) and left undisturbed to habituate to their environment for 2 days. Continuous recording started 2 days before the first administration (baseline). For the study with almorexant, to save on telemetry transmitter battery life, data were then recorded 4 days (instead of 7 days) per week. Recording during the last week of treatment continued until the end of the 7 days of recovery. For the study with zolpidem, data were recorded continuously during the 5 days of treatment, stopped 2 days after the last administration from the first part of the crossover and restarted 2 days before the beginning of the administration of the second part of the crossover. Recordings were stopped 2 days after the last administration.

**Statistical analysis**

Data are presented as means ± SEM. Statistical analyzes were performed by two-way analysis of variance (ANOVA) using GraphPad Prism and Bonferroni post-hoc test for multiple comparisons. The null hypothesis was rejected when p < 0.05.
